# Engineering bacterial theranostics: from logic gates to *in vivo* applications

**DOI:** 10.3389/fbioe.2024.1437301

**Published:** 2024-09-18

**Authors:** Angus Armstrong, Mark Isalan

**Affiliations:** ^1^ Department of Life Sciences, Imperial College London, London, United Kingdom; ^2^ Imperial College Centre for Synthetic Biology, Imperial College London, London, United Kingdom

**Keywords:** synthetic biology, theranostics, prokaryotic gene circuits, logic gates, gene networks, microbiome and dysbiosis, tumour microbiome

## Abstract

Over the past 2 decades, rapid advances in synthetic biology have enabled the design of increasingly intricate and biologically relevant systems with broad applications in healthcare. A growing area of interest is in designing bacteria that sense and respond to endogenous disease-associated signals, creating engineered theranostics that function as disease surveyors for human health. In particular, engineered cells hold potential in facilitating greatly enhanced temporal and spatial control over the release of a range of therapeutics. Such systems are particularly useful for targeting challenging, under-drugged disease targets in a more nuanced manner than is currently possible. This review provides an overview of the recent advances in the design, delivery, and dynamics of bacterial theranostics to enable safe, robust, and genetically tractable therapies to treat disease. It outlines the primary challenges in theranostic clinical translation, proposes strategies to overcome these issues, and explores promising future avenues for the field.

## 1 Introduction

Due to both their commensal properties and the growing ease and complexity in which their genetic code can be altered, bacteria have emerged as a potential leading therapeutic chassis, with promise for targeting diseases ranging from rare metabolic diseases to cancer and diabetes (Ozdemir et al., 2018). Of particular interest are bacterial therapies that can detect signals and respond with designed behaviours. Such “sense-and-respond” therapies could act jointly as therapeutics and diagnostics (theranostics) and can be further subcategorized into closed-loop or open-loop therapies. Closed-loop therapies use feedback to self-regulate by detecting a biomarker and adjusting accordingly, while open-loop therapies lack self-regulation as they rely solely on external inputs (Cammack, 2006). By designing closed-loop theranostics, therapeutic release can be molecularly tethered to the disease target in time and space, responding autonomously to changes in disease progression, thus providing homeostatic regulation.

**FIGURE 1:**
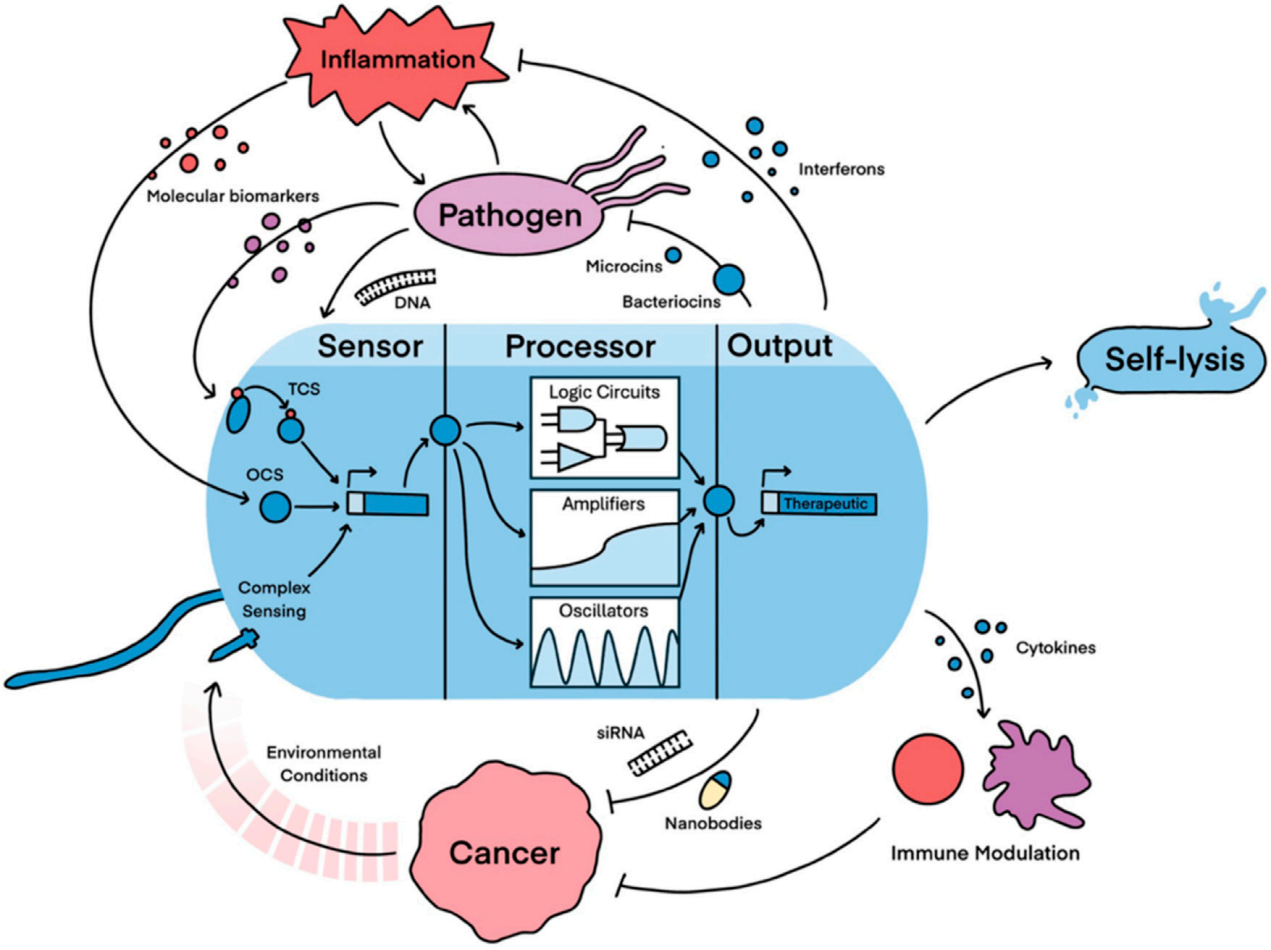
Illustration of the key components and promising functions of a bacterial theranostic. Theranostic sensors have been designed to detect a variety of molecular and environmental inputs predominantly via two-component systems (TCSs), one-component systems (OCSs) and complex sensing mechanisms. The processor module incorporates more complex genetic circuits to add sophistication to the device. This includes the design of Boolean systems to regulate expression (logic circuits), amplifying the expression of the output, and engineering oscillatory expression of the output. Most theranostic outputs are therapeutics, but self-lysis can also be triggered as an output to limit theranostic persistence, release large payloads, and facilitate the release of immunogenic adjuvants. Key therapeutic targets with growing potential are then shown. Cancer can be targeted directly via the release of various payloads or indirectly through immune modulation, whereas dysbiosis is primarily targeted by limiting pathogen outgrowth and reducing inflammation. These diseases are promising for bacterial theranostics because they provide many biotic and abiotic conditions that the theranostic can detect, hence allowing the design of closed-loop therapies.

In bacterial theranostic design, three primary components are typically present: a sensor module, processing module, and output module (Figure 1). The processing module can greatly increase the sophistication of a system and, while many synthetic bacterial gene circuits (Michalodimitrakis and Isalan, 2009) and conditional logic gates (Tamsir et al., 2011) have been designed, some of these motifs are particularly useful for therapeutics, as shown in Table 1. Together, these three modules can be engineered to create increasingly nuanced theranostics. Designing effective theranostics also often relies on harnessing natural bacterial behaviour. In this way, harnessing distinctive combinations of chemotaxis, tropism, and sensing systems, which are native and often unique to different chassis, can help facilitate more complex therapies.

**TABLE 1:** Key processing module circuit motifs applied to bacterial theranostics.

		Example Circuit Design	Function and Theranostic Use
Logic Gates	AND Chien et al. (2022)	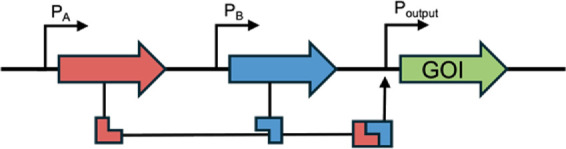	Both biomarkers A and B must be sensed for activation of a therapeutic response. This allows more specific disease condition detection
	OR Garrido et al. (2021)	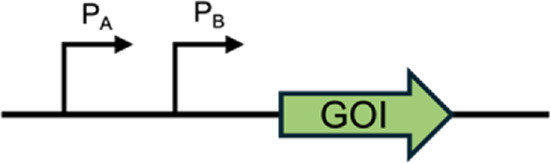	Multiple biomarkers can trigger the same therapeutic response. This allows broader disease condition detection
	NOR Chua et al. (2023)	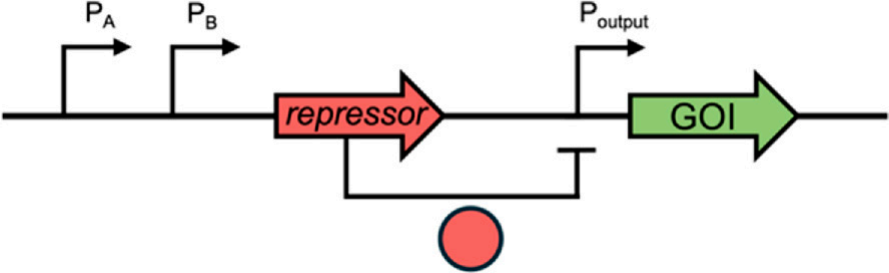	A low therapeutic response occurs when there are high levels of either biomarker. All other logic gates can be designed from NOR.
	XOR Najafi et al. (2021)	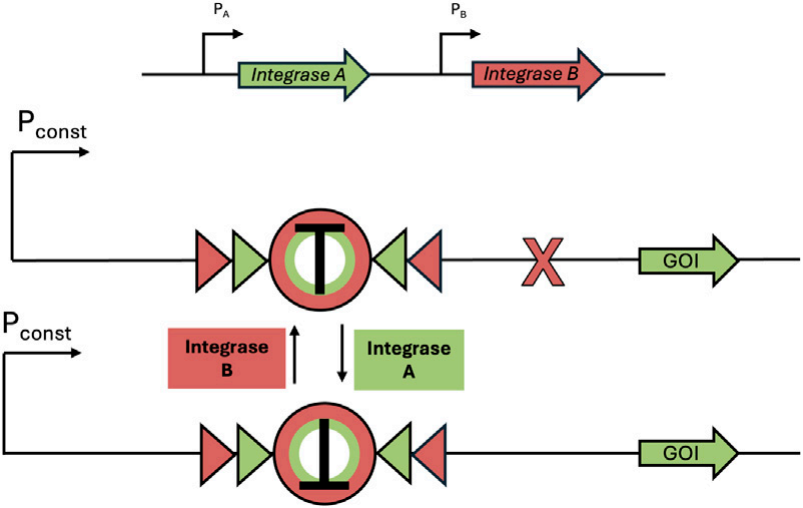	This gate gives an output when one biomarker is present but no response when both biomarkers (or neither) are present. In this example, a terminator (T), flanked by two sets of recombination sites, prevents transcription. Expression of an integrase flips T so that transcription can occur, but expression of a second integrase returns T to its correct orientation, preventing transcription. This gate is used in biocontainment and gene amplification
Memory	Base-editing Cheng et al. (2023)	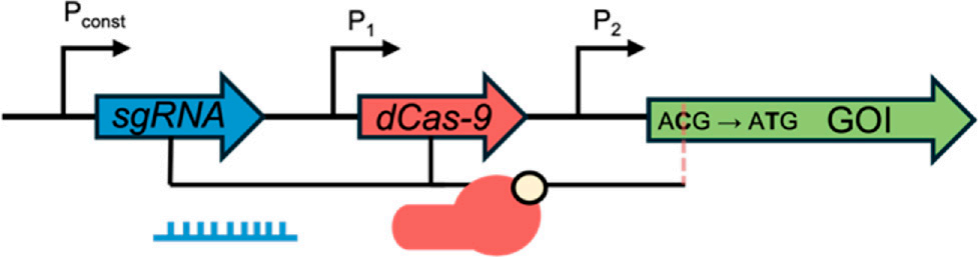	When induced, dCas-9 with a fused cytosine deaminase can single base-edit a C to a T, guided by constitutively expressed sgRNA. This creates a start codon that allows translation of the GOI. This acts as an inheritable genetic memory so that one can discern whether the theranostic sensed a biomarker after the input has stopped
	Toggle-switch Tica et al. (2024)	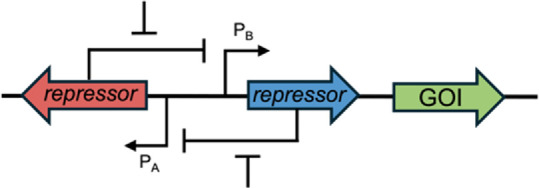	Transcriptional memory imparts the ability to discern whether the theranostic sensed a specific input after the input has stopped

PA/B, inducible promoter A/B; Pconst, constitutive promoter; GOI, gene of interest.

In this review, we will discuss the key design strategies and choices that should be addressed when designing closed-loop bacterial theranostics. Bacterial theranostics have been engineered to respond to a range of physiological changes, ranging from fluctuations in cortisol levels to the presence of toxic metals (Chen et al., 2023; Litteral et al., 2023). However, here we will concentrate on applications for diseases where the need for better therapies is most promising and pressing, specifically in targeting dysbiosis and cancer. Despite their potential, no closed-loop bacterial therapies have yet reached the clinic, therefore we shall analyse the challenges that must be addressed to facilitate the rational design of robust, sensitive and safe therapeutics.

## 2 Applications of engineered bacterial theranostics

### 2.1 Targeting gut dysbiosis

Because of its accessibility and propensity to host commensal bacteria, the gut is an attractive location for the targeted delivery of bacterial theranostics to treat dysbiosis. The gut environment is highly heterogeneous and varies both along its length and on its luminal depth. Progress over the last two decades has begun to uncover the vast network of microbial interactions and their influences on host biology. Indeed, gut microbes outnumber total human cell number 10 to 1, and genomic content 100 to 1 (Backhed et al., 2005; Thursby and Juge, 2017). These interactions are both spatially and temporally dynamic, and changes in them can lead to dysbiosis. Despite recent advances, the specific aetiologies of many gut-related diseases are still unknown but alterations in gut microbiota composition are thought to play a role in many, including inflammatory bowel disease (IBD), cancers, and autoimmune disorders (DeGruttola et al., 2016). Current treatments for many gut-related diseases are predominantly limited to: faecal microbial transplantation, which is often invasive and lacks standardisation; oral medications, whose efficacies are limited by drug inactivation and adverse side effects; or lifestyle changes, which have low compliance levels (Bull and Plummer, 2015). By contrast, bacterial theranostics could provide non-invasive, effective, and precise treatment options which promote eubiosis.

**FIGURE 2:**
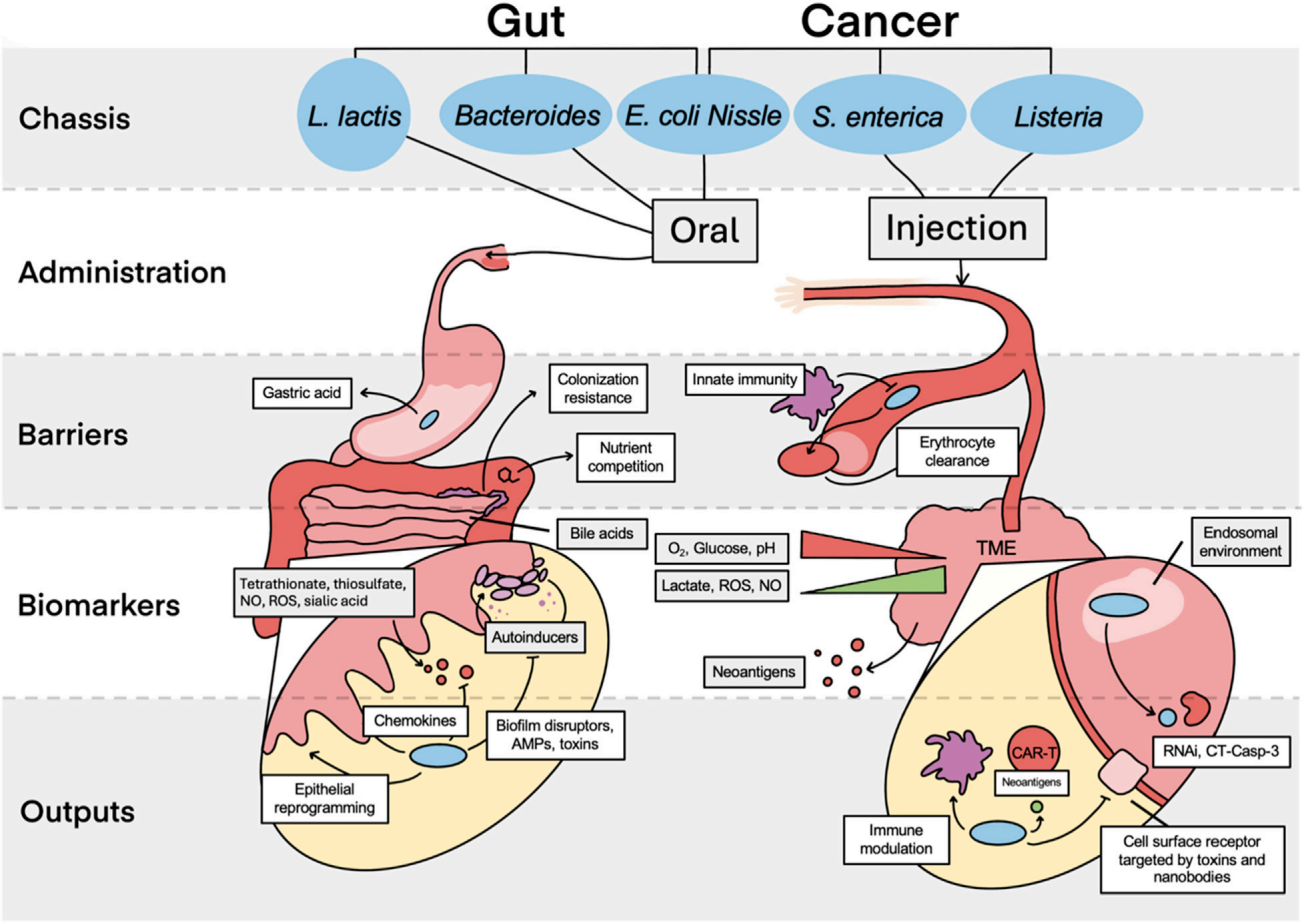
Comparison of gut- and tumour-targeting theranostics. The key chassis used for theranostic design are shown, with *E. coli* Nissle commonly used for both cancer and gut therapies. Administration is limited primarily to oral intake or injection, with injection possible intravenously or intratumorally. These two administration routes both have distinct barriers to reaching their target. Finally, assuming successful bacterial localisation, several of the key biomarkers and theranostic responses to these are illustrated for the gut and TME, showing magnifications of the colon epithelial layer and the interface between a cancer cell and surrounding extracellular matrix respectively. TME; tumor microenvironment, NO; nitric oxide, ROS; reactive oxygen species, AMP; antimicrobial peptides, RNAi; RNA interference, CT Casp-3; constitutive two-chain active caspase-3.

To produce effective and safe therapies against gut dysbiosis, theranostics must be able to integrate into a specific niche, where they robustly and sensitively deliver a payload in response to an environmental change (Figure 2). Using a probiotic bacterial chassis is therefore beneficial due to its evolved ability to sense within physiologically relevant parameters and safely colonize specific niches within the gut. Historically, *E. coli* Nissle 1917 (*E. coli,* EcN) was the primary chassis of choice, due in part to the large available genetic toolkit for *E. coli*. However, other chassis including *L. lactis* and *Bacteroides* are becoming more widely used and more easily engineered (Mao et al., 2018; Taketani et al., 2020). Whilst some native functions of these probiotic chassis are beneficial, others may be detrimental. For example, concern had been raised with the *pks* cluster in EcN and its association with colorectal cancer (Kalantari et al., 2023); in this case, the cluster was removed without impeding function.

Inflammation is a key hallmark of dysbiosis but to target inflammation, reliable biomarkers must be identified. Consequently, several molecular markers, such as nitric oxide (NO), tetrathionate, thiosulfate and sialic acid have been studied, from which theranostics can be designed (Table 2). For example, EcN has been engineered to release anti-inflammatory interferons in response to NO, promoting upregulation of suppressive transcription factors in regulatory T cells (Tregs). This in turn leads to increased epithelial structural integrity and anti-inflammatory cytokine levels (Chua et al., 2023).

**TABLE 2:** Recent examples of closed-loop bacterial theranostics.

Disease Target(s)	Biomarker(s)	Chassis	Sensing	Processing/Output	Administration Method	Safety measures	Host organism	References
Intestinal *P. aeruginosa*	3OC12HSL	EcN	*lasR-lasI* quorum-sensing system	Microcin V secretion pathway and YebF secretion peptide PA2-GNU7 *P. aeruginosa*-selective AMP secretion	Oral	No	Mice	Kim et al. (2023)
CDI	Sialic acid	EcN	*nanR-*pNanA	*cadC-*pCadBA amplifier, deconjugation of taurocholate to cholate by bile salt hydrolase (Cobh) reduces endospore germination, growth and toxin secretion	Oral	*Δalr ΔdadX* D-alanine auxotrophy	C57BL/6 mice	Koh et al. (2022)
Colorectal cancer, Gut Dysbiosis	L-lactate, H^+^, hypoxia	EcN, SBC	pLldR, pCadC and pPepT drive expression of serine integrase	XOR amplifier, release of hemolysin (HlyE)	ITI, Oral	*φX174E* expression causes self-lysis in TME environment	CT26 mice, AOM/DSS-induced mice	Zhou et al. (2024)
Colorectal cancer	H^+^	*E. coli* MG1655	pAdiA	*clyA/GFP* expression causes tumour cell perforation and death, endogenous inflammatory factors led to tumour blood vessel thrombosis, increased coagulation, and arrested tumour growth as well as reduced lung metastasis	IVI	Showed no signs of presence in other organs	CT26 mice	Qin et al. (2022)
Colorectal cancer	NO	*S. typhimurium*	NorR-pNorV	NO-sensing gene switch system through reorientation (FimE recombinase) of FimS driving *GFP* expression. Natural immunogenicity upregulated innate immune response	IVI	*ΔppGpp* attenuation reduced persistence and antibiotic resistance	Mice	Qin et al. (2023)
Breast Cancer and Liver Cancer	Endosomal environment	*Salmonella enterica*	Promoters of SPI2 genes	PBAD-*flhDC* controls cell invasion, CT Casp-3 anti-cancer protein secretion	ITI, IVI	pBAD-*lysE* self-lysis	Mice	Raman et al. (2021)
Cancer	Hypoxia	EcN	pFnrS	*dacA* expresses cyclic dinucleotide that activates STING in phagocytic APCs, natural immunogenicity upregulated innate immune response	IVI	*ΔthyA* and *ΔdapA* auxotrophy	Mice	Leventhal et al. (2020)
IBD	Thiosulfate	EcN	*thsS/R* TCS	Base-editing memory, fluorescent reporting, AvCystatin with fused signal peptide, stimulation of macrophage cytokines	Oral	No	DSS-induced mice	Zou et al. (2023)
IBD	NO and RNI	EcN	NorR-pNorV	YebF secretion tag with IFNL1 interferon	N/A	No	*in vitro*	Chua et al. (2023)
Elevated Cortisol	Mammalian cortisol	EcN	*C. scindens* LysR cortisol sensor	Tryptophan decarboxylase (*tdc*) expression increases serotonin and tryptamine levels, *ΔtolC*	N/A	No	*in vitro*	Litteral et al. (2023)
Skin MRSA	AgrD	*S. epidermidis*	*agrCA* quorum-sensing circuit (TCS)	P2/P3-lysostaphin	Topical	No	Mice	Guan et al. (2022)

EcN*, E. coli* Nissle 1917, SBC, synthetic bacterial consortium; IBD, irritable bowel disease; CDI, *C. difficile* infection; TCS, two-component system; SPI2, *Salmonella* Pathogenicity Island II; NHP, non-human primate; NO, nitric oxide; RNI, reactive nitrogen intermediates; ITI, intratumoral injection; IVI; intravenous injection, STING; simulator of interferon genes, APC; antigen presenting cell.

Circuits capable of generating multiple responses from a single sensor can enhance the sophistication of therapeutic interventions. For example, engineered EcN could secrete the immunomodulatory drug AvCystatin, alleviating IBD in mice by sensing elevated thiosulfate levels (Zou et al., 2023). Moreover, the strain produces a fluorescent reporter and encodes a memory via single-base editing, enabling detection of past inflammation levels even after their reduction. As well as engineering multiple outputs, multiplexed input systems have been designed to allow greater control over therapeutic release. For example, a system has been designed that uses an AND logic gate so that an output is only achieved in the presence of both thiosulfate and nitrate (Woo et al., 2020).

Other logic gates have been devised to respond to hallmarks of dysbiosis characterized by reduced biomarker concentration. Short chain fatty acids (SCFA), such as butyrate and propionate, are key multifunctional microbial metabolites found in the gut and exhibit anti-inflammatory properties (Mann et al., 2024). Researchers constructed a NOT gate system for both these SCFAs so that increased anti-inflammatory cytokines are released at reduced levels of SCFA (Serebrinsky-Duek et al., 2023).

Increasing engineered circuit complexity can lead to higher metabolic burden, so designing optimized circuits is important. Aided by the circuit automation software “Cello,” researchers designed a *Bacteroides thetaiotaomicron* (*B. thetaiotaomicron*) strain with location-specific control of expression with an XOR logic gate (Taketani et al., 2020). The strain used a bile acid sensor, anhydrotetracycline (aTc) sensor, and three output genes to control gene expression, depending on whether the strain is in the fermenter, gut, or elsewhere, thus providing built-in biocontainment.

### 2.2 Treating pathogens in the gut and beyond

As well as targeting inflammation to treat dysbiosis, theranostics can be designed to target and inhibit the outgrowth of specific pathogens. Through sensing tetrathionate, EcN was engineered to secrete microcin H47 which specifically targeted *Salmonella* and reduced infection (Palmer et al., 2018). On the other hand, pathogen growth can be inhibited indirectly by targeting a molecule required for pathogenic growth and survival. For example, *C. difficile* endospore germination is reliant on the presence of taurocholate, a bile salt conjugate that is upregulated in dysbiosis (Theriot et al., 2016). A recent study engineered EcN to respond to sialic acid by producing increased levels of bile salt hydrolase, which eradicated *C. difficile* infection in mice by lowering taurocholate levels (Koh et al., 2022).

Quorum signalling (QS) molecules are a mainstay of synthetic gene circuit engineering and are used to communicate between cells in some of the largest and most complex networks reported to date (Moon et al., 2012; Tica et al., 2024). If QS signals could be sensed, theranostics could be pathogen-specific rather than dependent on inflammatory signals. Recently, *P. aeruginosa* infections were treated in mice by engineering EcN that respond to the strain-specific autoinducer molecule 3OC12HSL (N-3-oxododecanoyl homoserine lactone) by secreting a hybrid antimicrobial peptide that specifically targets *P. aeruginosa* (Kim et al., 2023). Similarly, an earlier study targeted the same pathogen via the release of toxins and biofilm disrupters (Hwang et al., 2017).

A different chassis has been used to target cholera infection: a hybrid two-component system was engineered into the probiotic strain *L. lactis* by combining a *V. cholerae* autoinducer binding domain with an *L. lactis* signal transduction domain to specifically detect and suppress cholera infection in mice (Mao et al., 2018). This kind of modularity in two-component systems could be harnessed to target a wide range of molecules. Future techniques could even include detecting pathogenic DNA, such as the recent example of a biosensor for sensing multiplexed DNA from multiple pathogens (Cheng et al., 2023).

Whilst most current applications target dysbiosis of the gut, other targetable communities exist. For instance, dysbiosis of the skin contributes to many conditions, ranging from dermatitis to acne (Tomida et al., 2013; Byrd et al., 2017). The skin commensal *Staphylococcus epidermidis* (*S. epidermidis*) is a common chassis of choice for this organ, and naturally inhibits *Staphylococcus aureus* (*S. aureus*) biofilm formation and epithelial adhesion (Sugimoto et al., 2013). A study engineered *S. epidermidis* to sense autoinducers specific for methicillin-resistant *S*. *aureus* (MRSA) and release the bacteriocin lysostaphin in response to inhibit MRSA growth (Guan et al., 2022). However, its performance *in vivo* was not adequate, potentially due to an uneven distribution of theranostic on the skin. Similar therapies could help improve gynaecological health in the future. Indeed, this microbiome has only recently gained the attention it requires; a project is set to start soon that develops an engineered probiotic to treat recurrent bacterial vaginosis (Corcoran, 2023). Clearly, bacterial theranostics are applicable in a wide variety of physiological environments.

### 2.3 Targeting the solid tumour microenvironment

Solid cancers are a tumour subtype primarily originating from epithelia of solid organs such as the breast and colon (Najafi et al., 2021). These tumours are “under-drugged” and have significantly worse clinical outcomes compared to liquid tumours, comprising greater than 85% of all cancer-related deaths, so there is a growing need for novel effective solutions (Najafi et al., 2021). Different tumour microenvironments (TMEs) have distinct, primarily cancer-promoting bacterial communities (Nejman et al., 2020). Despite this, bacteria have also been used to treat cancer for over a century (Coley, 1991), and there has been growing interest in employing synthetic biology principles to further enhance their therapeutic potential. Given that bacteria can show strong TME tropism and penetrate deep into the tumour core, bacterial theranostics have the potential to target regions impenetrable by conventional therapies. Let us first consider the natural and engineered behaviour of bacteria to sense and respond to solid tumours.

Circulating bacteria are highly immunogenic which can make administration challenging (Najafi et al., 2021). However, bacteria engineered to evade innate immune detection can be injected into the bloodstream; whilst removing particularly immunogenic proteins like lipopolysaccharides does reduce systemic inflammation, it also reduces therapeutic activity (Toso et al., 2002). To mitigate this lowering of activity, bacteria could be specifically injected into the immune-privileged tumour vasculature or directly into the tumour. Nonetheless, is it possible that such injections might result in some leakage of the microbes into the bloodstream. Any microbes that are able to infiltrate into the bloodstream at low quantities are still likely to infiltrate into the tumour mass and thus perform their designated functions. Additionally, biosensing systems can be engineered to address administration safety without compromising on efficacy: a recent study demonstrated that engineering bacterial surface capsular polysaccharide (CAP) production to be regulated by isopropyl β-d-1-thiogalactopyranoside (IPTG) improved EcN localization to distal tumours (Harimoto et al., 2022). This allowed for higher bacterial dosages, with reduced clearance rates, to be administered, with CAP levels returning to baseline when IPTG was removed. Future modifications could enable endogenous factors to regulate this instead of IPTG, creating a self-tuneable protective layer for bacterial administration in the bloodstream.

The TME is characterized by highly specific environmental conditions including hypoxia, acidity, reduced glucose levels, elevated lactate levels, and increased reactive oxygen and nitrogen species (de Visser and Joyce, 2023). There has also been a recent focus on detecting cell-free cancer DNA (Cooper et al., 2023). Through the design of condition-responsive elements, therapies can be engineered to express anti-cancer therapeutics only under such conditions (Table 2). Inducible systems thus permit delivering payloads that would be toxic if introduced systemically within bacteria. These anti-cancer payloads are varied and include toxins, nanobodies, antigens and cytokines. Furthermore, non-invasive microbes like EcN are often used which, unlike other chassis, show no colonization of healthy tissue (Stritzker et al., 2007). External payload delivery allows bacteria to proliferate to higher colony numbers for higher payload concentrations. Moreover, QS can be harnessed to coordinate therapeutic release, limit release from bacteria occupying healthy tissues in small numbers, and upregulate the innate immune system. An important study demonstrated that cyclic release of a nanobody antagonist, regulated by QS, led to multifaceted anti-cancer improvements in mice through lysis of engineered EcN targeting an overexpressed anti-phagocytic receptor in human cancers (Chowdhury et al., 2019). Coordinated lysis of this therapy also released components that acted as adjuvants for the innate immune system. In the future, the sequential release of different payloads may thus be possible through QS signalling.

Many hallmarks of cancer are regulated via intracellular pathways, but targeting these with standard chemotherapies is challenging. The invasive properties and intracellular lifecycle of *Salmonella enterica* serovar *typhimurium* (*S. typhimurium*) can be harnessed to target these pathways. This strain has been designed to release an apoptosis-initiating drug into the cytoplasm before self-lysing. The approach not only reduced breast tumour size ∼2-fold in triple-negative breast cancer model mice but was also effective against two liver cancer models (Raman et al., 2021). When treating BNL-MEA tumors in C57L/J mice, the engineered *S. typhimurium* was able to reduce tumour growth by almost ∼60% relative to Sorafenib, which is the standard treatment for liver cancer. Furthermore, lifespan was significantly prolonged in Hepa 1–6 tumours: untreated control mice were all dead after 30 days, whereas ∼25% of treated mice were alive after 100 days. Impressively, one treated mouse remained disease-free after 300 days, indicating a functional clearance of the tumour (Raman et al., 2021). The sensing machinery required to orchestrate this system is complex and involves flagella and a Type-III secretion system, highlighting the current necessity in theranostic top-down approaches that consider the whole cell properties. *S. typhimurium* has also very recently been engineered to inhibit the key oncogene regulator c-Myc through RNA interference *in vivo* (Williams et al., 2024).

The natural immunogenicity of bacteria can itself have a therapeutic benefit through upregulation of the host immune system around the TME. Synthetic engineering approaches have also allowed specific steps in the innate or adaptive immune responses to be targeted. STimulator of INterferon Genes (STINGs) play a key role in innate immune responses to intracellular pathogens (Zhu et al., 2019). By engineering hypoxia-driven circuits in EcN to release STING agonists, and harnessing the natural activation of pattern-recognition receptors, Leventhal *et al.* increased the production of interferons around tumours, which increased anti-tumour immunity (Leventhal et al., 2020). Recent research has used the same strain to express the chemokine CXCL16 which recruited activated T cells to the tumour site, increasing adaptive immune responses. The same paper also showed that the innate immune response was activated by recruitment of dendritic cells through chemokine CCL20 expression. Together, these two bacteria showed synergistic anti-tumour functions when administered simultaneously (Savage et al., 2023).

Finally, using a probiotic chassis to treat colorectal cancer has shown multifaceted benefits both in tumour suppression and reducing dysbiosis, which is often dysregulated by chemotherapy (Huang et al., 2023). An EcN consortium was engineered to release a therapeutic payload of hemolysin under TME conditions of hypoxia, low pH, and high lactate levels, to target colorectal cancer, implementing an XOR amplifier (Zhou et al., 2024). This study addresses challenges related to administration, tumour tropism, payload delivery, and controlled release, and points the way forward in applying synthetic biology concepts, such as logic gate engineering, to bacterial theranostics.

## 3 Challenges in robust biocontainment and biosafety

**FIGURE 3:**
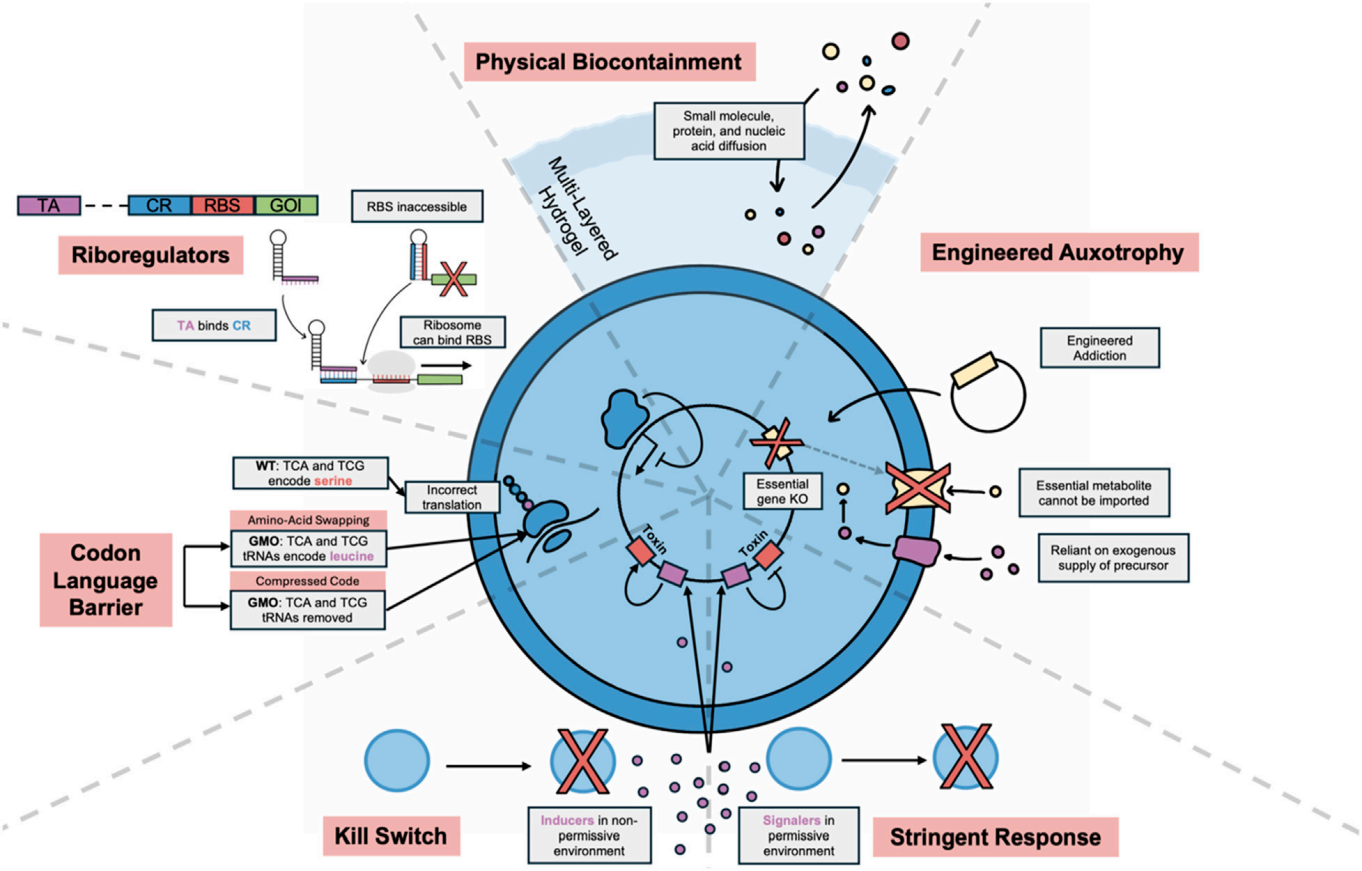
Overview of key biocontainment strategies. Physical biocontainment techniques allow molecular communication but keep cells physically separated from environment. Multilayered hydrogels allow for better biocontainment, with a harder outer shell to protect from physical stress. Engineered auxotrophy allows survival only in the presence of an essential molecule which the bacteria cannot utilise or synthesise. This can be used to engineer addiction to a heterologous plasmid by expressing the essential gene on the plasmid. Kill switches cause cell death in the presence of an inducer from a non-permissive environment, whereas stringent response systems encode a toxic gene that is maintained “OFF” until a signal molecule is lost. Codon language barrier techniques include engineering swapped codons for amino acids and compressed codes. These methods limit biocontainment because GMO mRNA will be incorrectly translated from non-GMOs. Riboregulators can aid in biocontainment by limiting partial DNA transfer. The CR binds to the RBS, preventing ribosomal binding and translation. The distant TA competitively binds to the CR, which makes the RBS accessible to the ribosome. If the TA is not taken up with the CR, gene expression will be repressed in non-GMOs. KO; knock-out, GMO; genetically modified organism, RBS; ribosomal binding site, TA; transactivating RNA, CR; cis-repressing RNA.

Ensuring biocontainment remains a primary challenge, hindering clinical translation of bacterial theranostics. Encapsulation with hydrogel materials is a physical biocontainment method that permits bacterial sensing and growth while preventing direct contact. This approach has been used to enhance survival in the gut and to reduce bacteraemia in the bloodstream (Hamidi Nia and Claesen, 2022). A recent study showed that alginate encapsulation greatly increased the recovery of thiosulfate-sensing EcN from the guts of rats and accurately responded to the colitis disease state (Aghlara-Fotovat et al., 2023). However, physical techniques are accompanied with intrinsic risk of failure and prevent interaction of bacteria with their environment, so engineered biocontainment through targeting molecular mechanisms is essential for safe therapeutic design. These strategies are large in number and Figure 3 covers the key approaches and promising advancements. Recently, both bacterial horizontal gene transfer (HGT) and viral infection were prevented in bacteria with swapped amino acid code, because incorrect primary protein sequences are translated outside of the engineered genetic code (Nyerges et al., 2023).

Biocontainment strategies that only regulate one target are at higher risk of loss-of-function mutations. To address this, creating efficient feedback loops that tune heterologous expression levels could reduce metabolic burden and thus mutation rates (Huang et al., 2024). An alternative solution to this problem is to implement multiple strategies within one chassis. Exemplifying this approach, Hayashi et al. (2024) combined engineered auxotrophy, kill switches and riboregulator-controlled expression to produce a multi-level, robust biocontainment strategy for *B. thetaiotaomicron*. More complex biocontainment strategies can also improve therapeutic function: Chien et al. (2022) created a multiplexed biosensor that uses an AND logic gate to drive an essential gene. This strategy improved the bacterial response to physiological cues, whilst limiting growth to defined environments, with different strains engineered for the gut and TME.

Other systems have also avoided single factor strategies for biocontainment, for example, in *Mycoplasmas*. These bacteria are challenging systems for engineering and have been used for eliminating *S*. *aureus* biofilms *in vivo* (Garrido et al., 2021). In this chassis, double kill switches have been designed that decrease the escape frequency below the National Institutes of Health (NIH) recommended threshold for GMOs (<10^–8^) (Broto et al., 2022). While many systems have successfully limited escape rates, implementing and testing these in bacteria under the burden of producing heterologous therapies must become commonplace in pre-clinical research.

Another exciting area of progress is in the site-specific incorporation of noncanonical amino acids into both prokaryotic and eukaryotic proteins (Chin, 2014). This is done by altering translation through the incorporation of an orthogonal aminoacyl tRNA synthetase and tRNA pair and has a multitude of potential applications, which include improving biocontainment. For example, simple auxotrophy biocontainment strategies can be escaped through several mechanisms and so Mandell et al. (2015) engineered an essential gene to contain a noncanonical amino acid at a location necessary for its function. This successfully protected against escape mutations, horizontal gene transfer and alternative metabolite utilisation, because bacterial survival was now dependent on the presence of an exogenously supplied metabolite that was not present naturally in the environment. Furthermore, synthetic auxotrophy has been shown to very strongly limit escape from batch cultures using continuous evolution and also showed robust containment within a mammalian culture (Kunjapur et al., 2021). Importantly, protocols have also been developed for the efficient incorporation of several noncanonical amino acids into functional proteins of the gram-positive probiotic *Bacillus subtilis* (Stork et al., 2021). However, there is still much improvement required before cheap and robust incorporation of unnatural amino acids for biocontainment is possible in a variety of useful chassis. Along with their potential in biocontainment, noncanonical amino acids have therapeutic uses, as shown in a recent paper where an engineered anti-cancer drug, containing an unnatural hydrophobic amino acid, increased its uptake and accumulation in immunosuppressive cancer cells (Yi et al., 2024).

As well as great heterogeneity within an organism, physiological parameters in diseased states can vary significantly between individuals, so a therapy that is well-tuned to one individual may be less so in another, resulting in increased escape rates. Therefore, it may be beneficial to pursue more personalised therapeutic avenues, requiring bacterial theranostics to be reprogrammed and retuned to tailor therapies for different patients. This has been successfully achieved in the Collins laboratory, especially in the creation of the ‘Passcode’ biocontainment circuits, where an essential gene is only expressed in the presence of inputs A and B, but in the absence of input C, where these inputs can be varied (Chan et al., 2016). Furthermore, many of the logic gates described earlier in the paper for therapeutic applications could be altered to also control the expression of an essential gene in future experiments.

## 4 Conclusion and Outlook

Despite recent progress, the functionality of single chassis theranostics is limited by resource competition and metabolic crosstalk (Jia et al., 2016). By sharing functions across strains, synthetic bacterial consortia could reduce metabolic load whilst allowing different chassis to be better suited to different functions. This allows not only for network engineering at the genomic level but also at the community level. Although all 16 two-input Boolean logic gates have been designed in multicellular systems, enhancing specificity and minimising output leakage is crucial for therapeutic applications (Tamsir et al., 2011). The ability to target individual strains for genetic manipulation within a consortium is also essential, with recent work providing a computational design of strain-specific guide RNAs for CRISPR gene editing (Rottinghaus et al., 2023).

As well as engineering bacterial communities, cross-kingdom communities are beginning to be designed, most notably in the use of bacteria to improve chimeric antigen receptor T-cell (CAR-T) therapy in solid cancers. Solid cancers are more challenging targets for CAR-T cells due to the lack of conserved tumour restricted antigens, which present significant risk of off-target, fatal toxicity in healthy tissues (Parker et al., 2020). CAR-T tumour targeting was recently improved by engineering orally administered EcN to release neoantigens into the extracellular matrix surrounding cancer cells (Vincent et al., 2023). Consortia of bacteria and viruses also hold potential, for example, with tumour-homing bacteria being used as vectors for oncolytic virus delivery (Sun et al., 2022). Furthermore, the potential in combining living and artificial cells is growing, with great progress in designing hydrogel-based biomimetic cells which perform basic cellular behaviours, including the release of a payload from subcellular compartments in response to an enzymatic biomarker (Allen et al., 2023). The integration of artificial and bacterial cells to produce biohybrid cells could address many challenges with bacterial therapies by reducing the need for biocontainment, reducing metabolic burden, and presenting fewer issues with biocompatibility (Elani, 2021).

As genetic engineering becomes more streamlined, the need for high-throughput, accurate methods to test bacterial functions increases. Progress in designing *in vitro* models that better mimic the complex *in vivo* environments will help facilitate this. For example, novel scaffolds have allowed a complex 3D model of the gut microbiota to be created which allows for biofilm formation (Calvigioni et al., 2023). Gut-on-a-chip design can allow for further improved mimicry of the physical stressors within the gut (Zhang and Qiao, 2023). Expanding the number of biologically relevant sensing circuits is also a key bottleneck in theranostic design. A recent paper addresses this by presenting a platform to allow rapid screening of novel biosensors through use of a bistable memory switch (Robinson et al., 2023). As well as screening new microbes, the recent growth in the power of directed evolution techniques could be harnessed to alter sensitivity or detect orthogonal inputs. Taken together, there is an exciting future ahead, where bacterial theranostics could revolutionize the treatment of disease, providing better outcomes for patients and safer therapies for the population.
